# High Levels of IL-18 and IFN-γ in Chronically Inflamed Tissue in Chronic Granulomatous Disease

**DOI:** 10.3389/fimmu.2019.02236

**Published:** 2019-10-18

**Authors:** Virginia Meda Spaccamela, Rocio G. Valencia, Oleksandr Pastukhov, Andrea Duppenthaler, Matthias S. Dettmer, Juliane Erb, Urs C. Steiner, Sven Hillinger, Carsten Speckmann, Stephan Ehl, Janine Reichenbach, Ulrich Siler

**Affiliations:** ^1^Division of Immunology, University Children's Hospital and Children's Research Center, Zurich, Switzerland; ^2^Zurich Center for Integrative Human Physiology, University of Zurich, Zurich, Switzerland; ^3^Institute for Regenerative Medicine, University of Zurich, Zurich, Switzerland; ^4^Unit of Pediatric Infectious Diseases, University Children's Hospital Bern, Bern, Switzerland; ^5^Institute of Pathology, University of Bern, Bern, Switzerland; ^6^Center for Dentistry, University of Zurich, Zurich, Switzerland; ^7^Department of Clinical Immunology, University Hospital Zurich, Zurich, Switzerland; ^8^Department of Thoracic Surgery, University Hospital Zurich, Zurich, Switzerland; ^9^Faculty of Medicine, Center for Chronic Immunodeficiency (CCI), Medical Center - University of Freiburg, Institute for Immunodeficiency, University of Freiburg, Freiburg, Germany; ^10^Faculty of Medicine, Center for Pediatrics and Adolescent Medicine, Medical Center, University of Freiburg, Freiburg, Germany; ^11^Center for Applied Biotechnology and Molecular Medicine, University Zurich, Zurich, Switzerland

**Keywords:** chronic granulomatous disease, hyperinflammation, macrophage priming, macrophage re-priming, IL-18/IFN-γ loop

## Abstract

**Background:** Chronic granulomatous disease (CGD) is caused by a malfunctioning nicotinamide adenine dinucleotide phosphate (NADPH) oxidase complex in phagocytes, leading to impaired bacterial and fungal killing and hyperinflammation.

**Objective:** To characterize macrophage subsets and cytokine/chemokine signaling loops involved in CGD tissue hyperinflammation.

**Methods:** Cytokine/chemokine production and surface marker expression were analyzed in inflamed tissue of four CGD patients and compared to cytokine/chemokine released by CGD macrophages upon priming to different macrophage subpopulations. Furthermore, the re-priming capacity of CGD pro-inflammatory M1 to M2a anti-inflammatory macrophages was evaluated.

**Results:** In human CGD inflammatory tissue, IL-18 and IFN-γ were detected in significant quantity. Immunofluorescence analysis identified macrophages as one source of IL-18 in inflamed tissue. *In vitro*, CGD macrophages could be primed and re-primed into all inflammatory/anti-inflammatory macrophage subpopulations. IL-18 was also released by M1 CGD and control macrophages.

**Conclusion:** CGD pro-inflammatory M1 macrophages remain M1 primed *in vivo*. As CGD M1 macrophages can be re-primed to anti-inflammatory M2a phenotype *in vitro*, macrophages are kept in M1 state *in vivo* by a persistent pro-inflammatory environment. Our results suggest a paracrine signaling loop between M1 macrophage derived IL-18 and non-macrophage derived IFN-γ maintaining macrophage pro-inflammatory activity in CGD tissue.

## Introduction

Chronic granulomatous disease (CGD) is a primary immunodeficiency caused by defects of the phagocyte nicotinamide adenine dinucleotide phosphate (NADPH) oxidase and concomitantly impaired generation of reactive oxygen species (ROS) (1, 2). CGD patients suffer from a predisposition to recurrent life-threatening fungal and bacterial infections, as pathogens can be engulfed by CGD phagocytes, but cells are unable to kill phagocytosed pathogens (3, 4). Pathogen-loaded CGD phagocytes form a barrier to antibodies and extracellularly acting antibiotics. The resulting infectious foci stimulate granuloma formation, partly through release and persistence of chemoattractants, which require oxygen metabolism for their degradation (5, 6). Another important manifestation is an enhanced and persistent inflammatory response. CGD patients may have concomitant autoimmune complications (7, 8), such as colitis (8–10). Up to 50% of CGD patients have gastrointestinal (GI) manifestations of their disease that include colitis and gastric outlet obstruction (9, 11, 12). The pathophysiology of CGD hyperinflammation is unclear. CGD patients present an increase of Th17 cells and Th17-derived cytokines (13), as well as high levels of serum antibodies to antigens present on gastrointestinal (GI)-tract associated microbes (14). Both increased pro-inflammatory activity and defective apoptosis of polymorphonuclear leukocytes (PMNs) have been linked to granuloma formation in X-CGD patients (15). Increased caspase 1 activation and IL-1β were reported in CGD macrophages after Nod-Like Receptor Pyrin 3 (NLRP3) inflammasome stimulation (16). Stimulation of CGD whole blood with *Aspergillus fumigatus* led to increased release of TNFα, IL-6, and IL-10, suggesting a dysregulation of pro-inflammatory and anti-inflammatory response (17). Clinical improvements of colitis in CGD patients were reported upon IL-1 blockage (18) and upon thalidomide treatment which led to suppression of TNF-α induced NFκB activation, a decreased bowel inflammation characteristic and reduced serum cytokines (19). Patients with CGD also present inflammatory conditions in the absence of detectable infections (20). Death of a CGD patient can be caused by an infection and/or by an inflammatory disease. Brown and colleagues reported on four CGD patients, of which two died during the course of the study. One patient died due to an infection and the other due to an overwhelming and irreversible pulmonary inflammation with no detectable infection (21). Macrophages play a pivotal role in tissue homeostasis and in the inflammatory process. Classically, macrophages and dendritic cells were seen to derive from a Macrophage Dendritic Cell Precursor (MDP) upon differentiation of monocytes in the steady state (22). Mouse studies revealed that tissue macrophages derive from embryonic precursors which already before birth seed the tissue and which in adult tissue maintain their number by self-renewal (23). The corresponding hypothesis that human tissue macrophages emerge from embryonic precursors is reasonable, but difficult to test. Most reports on mouse macrophages are conducted with bone marrow-derived macrophages (BMDM) and most human macrophage work was carried out with blood monocyte-derived macrophages.

Macrophages show high plasticity. Responding to stimulation, macrophages can shift their activity in a process termed polarization or priming. The distinction of mouse macrophages between classically activated (IFN-γ and LPS) M1 macrophages with pro-inflammatory phenotype and with pathogen-killing abilities, and alternatively activated M2 macrophages promoting cell proliferation and tissue repair (24) goes back to the initial work of Mills et al. (25). A further subdivision of M2 macrophages into M2a, M2b, and M2c was described by Mantovani et al. (26). M2a macrophages polarized upon exposure to IL-4 and dexamethasone (27) and M2b macrophages primed by immune complexes and LPS (28), were described to possess immunoregulatory functions and to drive type II responses, whereas exposure of macrophages to M-CSF and IL-10 (29) prime to M2c macrophages involved in immune suppression and tissue remodeling (26).

In healthy individuals, termination of a bacterial or a fungal infection is initiated by granulocytic ROS production triggering intra- or extracellular pathogen killing (30), as well as granulocytic cell death, leading to exposure of oxidized phosphatidylserine (PS) membrane lipids on cell debris (31). Macrophages recognize oxidized PS and efferocytose the cell debris, followed by induced IL-4 expression, secretion and self-stimulation of macrophages and termination of inflammation (32). In murine CGD models, an impaired efferocytosis and IL-4 expression were noted, together with an impaired invariant natural killer T (iNKT) cells activation, resulting in persistent M1 pro-inflammatory status of macrophages (32, 33). Treatment of CGD mice or *ex vivo* treatment of human macrophages with proliferator-activated receptor γ (PPAR-γ) agonists induced mitochondrial superoxide production and restoration of efferocytosis, thus bypassing an impaired NADPH oxidase activity (34, 35).

Efferocytosis of granulocytic debris and IL-4 self-stimulation induces a shift in macrophage priming from pro-inflammatory M1 into anti-inflammatory M2a status. Macrophages of X-CGD mice were reported as primarily M1-primed, whilst wild type (WT) macrophages were M2a-primed (32, 33). As the reason of the persistent pro-inflammatory state in CGD is only partially known, we set out to characterize primary-derived inflamed CGD tissue samples as well as *ex vivo* priming of CGD macrophages.

## Materials and Methods

### Patients

Heparinized blood samples and biopsies were obtained in compliance with local ethical requirements (ethical approval number KEK-ZH-Nr 2015-0135 and BASEC-Nr. PB_2016-0220) after obtaining written informed consent from healthy controls and CGD patients. Only blood samples of diagnosed CGD patients without residual NADPH oxidase activity who were not under steroid or IFN-γ treatment, and who were in a stable steady state without ongoing inflammation, were analyzed.

Patient 1 (acute dental abscess, 48 h) was a 6-year-old boy, diagnosed with X-CGD (*CYBB* c.632T > G) by molecular biology analysis (Sanquin, Amsterdam, The Netherlands) without detectable residual ROS formation by phagocytes in dihydrorhodamine (DHR) and nitroblue tetrazolium (NBT) tests. The patient presented with a dental abscess 48 h after minor trauma. At the time of the analysis, patient was otherwise asymptomatic, without clinical signs of inflammation at other body sites. He was under prophylactic treatment with itraconazole and co-trimoxazole, but did not receive IFN-γ or corticosteroids. Microbiological analysis of the abscess was not performed.

Patient 2 (subcutaneous abscess right side of the neck, 2 weeks) was a 16-year-old male diagnosed with p47phox-deficient CGD (*NCF1*, homozygous GT dinucleotide deletion in exon 2) by molecular biology analysis (Sanquin, Amsterdam, The Netherlands). Four percent of the patient's phagocytes possessed residual ROS activity (DHR assay). In the DHR assay, the shift in fluorescence intensity (ΔGeoMean MFI = GeoMean of mean fluorescent intensity (MFI) of PMA stimulated DHR-positive cells minus GeoMean MFI of unstimulated cells) of patient cells corresponded to 1.1% of the shift observed in a healthy control indicating a low level of residual cellular ROS production. The subcutaneous abscess at the right side of the neck was surgically drained (no microorganisms could be identified), followed by a course of antibiotic treatment, in addition to the standard prophylaxis with itraconazole and co-trimoxazole.

Patient 3 (sub-muscular thoracic abscess, 4 weeks), was a 27-year-old male, diagnosed with X-CGD at the age of 5 (*CYBB*). Patient presented with sub-muscular abscess of the thoracic wall, which was not connected with intra-abdominal or intra-thoracic walls. Histologically, the abscess was described as a mixed extended chronic granulizing and florid granulocytic, necrotizing and giant cell inflammation around few fungal elements. Microbiological analysis identified *Aspergillus fumigatus*. At the time of the analysis, patient was treated with tazobactam and voriconazole.

Patient 4 (submandibular lymph node abscess, 6 weeks) was a 1-year-old girl with AR CGD (*NCF1* delta GT). She was diagnosed with a bilateral submandibular fistulising lymph node abscess. Surgical drainage revealed the presence of *Burkholderia cepacia*. DHR test showed 17–25% of cells with residual NADPH oxidase function on a low level, as the shift of DHR-positive cells (ΔGeoMean MFI) corresponded to only 1.9% of the shift observed in healthy control cells.

A deep frozen control lymph node sample of a non-CGD patient presenting recurrent lymph node hyperplasia was kindly provided by the tissue biobank of the Institute of Pathology and Molecular Pathology of University Hospital Zürich.

### Flow Cytometry

Tissues were transported and homogenized as described in the Materials and Methods section “cytokine and chemokine analysis.” To avoid sampling errors, >50% of tissue was processed for flow cytometry analysis and cytokine quantification. Tissue-derived cells and *ex vivo*-primed macrophages were blocked with FcR blocking reagent (MACS, Miltenyi Biotec GmbH, Bergisch Gladbach, Germany) and stained with the antibodies as follows. Anti-human CD14 PC7 (clone RMO52), anti-human CD209 (DC-SIGN)-PE (clone AZND1), anti-human CD14 PC7 (clone RMO52) were from Beckman Coulter International S.A., Nyon, Switzerland. Anti-human CD15 V500 (HI98), anti-human CD57 FITC (HNK-1), anti-human CD56 PE (B159 RUO), anti-human CD8 V450 (RPA-T8) and anti-human CD8 V450 (RPA-T8) were from BD Bioscience, Eysins, Switzerland. Anti-human CD3 Alexa Fluor® 700 (UCHT1), anti-human Vα24Jα18 TCR PE-Cyanine7 (6B11), and anti-human CD206 (MMR) eFluor® 450 (clone 19.2) were from eBioscience, Eysins, Switzerland. Anti-human CD4 PerCP (OKT4), anti-human CD19 Brilliant Violet 605 (HIB19), anti-human CD163 PerCP/Cy 5.5 (clone GHI/61) anti-human CD86 Brilliant Violet 605^TM^ (clone IT2.2) were from BioLegend, Lucerna-Chem AG, Luzern, Switzerland. Anti-human Mer APC (clone 125518) were from R&D Systems, Bio Techne AG, Zug, Switzerland. Cells were washed, fixed with 2% formaldehyde in PBS, measured by GalliosTM flow cytometer and analyzed by Kaluza software (Beckman Coulter International S.A., Nyon, Switzerland).

### Cytokine and Chemokine Analysis

Volume of pus samples were determined and diluted to separate cells from supernatant by centrifugation. Solid tissues were kept in PBS on ice for up to 1 h until >50% of samples were weighed and homogenized with a syringe piston on a Petri-dish. Remaining tissue was removed by filtration (70 μm cell strainer). For flow cytometry analysis, cells were separated from supernatant by centrifugation. Supernatant was stored at −80°C until cytokine quantification in duplicates by 18plex Immunoassay (Cat. No. EPX180-12165-901, eBioscience, Thermo Fisher Scientific AG, Basel, Switzerland) and MILLIPLEX® MAP KIT (Cat. No. HCYTOMAG-60K, MILLIPORE, Merck AG, Zug, Switzerland) according to manufacturers' instructions for quantification of G-CSF, GM-CSF, IFN-γ, MDC, TNFα, IL-1β, IL-2, IL-4, IL-5, IL-6, IL-9, IL-10, IL-12p70, IL-13, IL-17A, IL-18, IL-21, IL-22, IL-23, and IL-27. Cytokines concentrations are expressed in ng/ml of undiluted tissue.

### Immunofluorescence

Paraffin-embedded slides were dewaxed and stained with rabbit anti-human IL-18 (LS-C313164, Lifespan Biosciences, LabForce AG, Muttenz, Switzerland) and mouse anti-human CD68 (clone 514H12, Novocastra Laboratories Ltd., Newcastle upon Tyne, UK), followed by donkey anti-rabbit FITC (711-095-152, Jackson ImmunoResearch Ltd., MILAN ANALYTICA AG, Rheinfelden, Switzerland), donkey anti-mouse Cy3 (715-165-150, Jackson ImmunoResearch Ltd., MILAN ANALYTICA AG, Rheinfelden, Switzerland) followed by mouse anti-FITC Alexa488 (200-542-037, Jackson ImmunoResearch Ltd., MILAN ANALYTICA AG, Rheinfelden, Switzerland) and 4′,6-Diamidin-2-phenylindol (DAPI) staining conducted by Sophistolab AG (Muttenz, Switzerland).

Slides were analyzed by confocal microscopy (model TCD SP8, Leica Geosystems Holdings AG, Glattbrugg, Switzerland) with original magnification x40. Acquired images were processed using ImageJ (36).

### Immunohistochemistry

Immunohistochemistry was perfomed by Sophistolab AG (Muttenz, Switzerland) on Leica BondMax instruments using Refine HRP-Kits (Leica DS98000, Leica Microsystems Newcastle Ltd., Newcastle upon Tyne, UK) according to manufacturer's instructions. Paraffin-slides were dewaxed, penetrated, and stained with rabbit anti-human IFN-γ (LS-B7487, Lifespan Biosciences, LabForce AG, Muttenz, Switzerland), visualized by HRP Refine Kit (DS9800, Leica Microsystems GmbH, Wetzlar, Germany). Images were then analyzed with a bright-field microscope (Axiovert S100TV, Carl Zeiss Vision Swiss AG, Feldbach, Switzerland).

### Monocyte Isolation and Differentiation to Macrophages

Monocytes were isolated from mononuclear cells after FICOLL (Ficoll-Paque PLUS, GE Healthcare AG, Glattbrugg, Switzerland) density gradient centrifugation, followed by magnetic bead-based positive selection (CD14 human microbeads, Miltenyi Biotec GmbH, Bergisch Gladbach, Germany) according to manufacturer's description. Cells were cultured (2.8 × 10^5^ cells/well) in PRIMARIA 24 wells plate (Becton Dickinson AG, Allschwil, Switzerland) in RPMI supplemented with Octaplas SD 10% (Octapharma AG, Lachen, Switzerland), 2 mM sodium pyruvate, 2 mM L-glutamine, 10 mM HEPES, and M-CSF 100 ng/ml (research grade, MACS, Miltenyi Biotec GmbH, Bergisch Gladbach, Germany) for 7 days.

### 24 h Priming of Macrophages

Macrophages were either kept in culture for 24 h in a fresh differentiation medium (M0 macrophages), or stimulated with IFN-γ (50 ng/ml, e-Bioscience, Thermo Fisher Scientific AG, Basel, Switzerland) and LPS (10 ng/ml, Sigma-Aldrich GmbH, Buchs, Switzerland) for M1 priming, IL-4 (20 ng/ml, ProSpec-Tany TechnoGene Ltd., Ness Ziona, Israel) for M2a, in wells pre-coated with human IgG (5 μg/cm^2^, Sigma-Aldrich GmbH, Buchs, Switzerland), and supplemented with LPS (100 ng/ml, Sigma-Aldrich GmbH, Buchs, Switzerland) for M2b, or supplemented with IL-10 (100 ng/ml, Sanofi Genzyme, Vernier, Switzerland) and M-CSF (100 ng/ml, MACS, Miltenyi Biotec, GmbH, Bergisch Gladbach, Germany) for M2c. For cytokine/chemokine analysis, supernatants were centrifuged, and aliquots were stored at −80°C. After 24 h of stimulation, macrophages were detached with EDTA and analyzed by flow cytometry analysis.

### Macrophage Re-priming

Supernatants from 24 h un-primed M0, or from 24 h M1, M2a, and M2c primed macrophages were removed, cells were washed with PBS without detachment, and re-primed for 72 h by incubation with priming stimuli (described in 24 h Priming of Macrophages). Ninty six hours after initial priming corresponding to 72 h after repriming, supernatants were centrifuged and stored at −80°C for cytokine/chemokine quantification.

### Statistical Analysis

Statistical analysis was performed as indicated by ANOVA with *post hoc* Bonferroni correction using SPSS (Version 22, IBM), and graphs were prepared using GraphPad Prism Software (Version 5.0a; GraphPad Software, La Jolla, California, USA).

## Results

### Characterization of Cellular Infiltrates in Acute vs. Chronically Inflamed Tissue

Biopsies of inflammatory lesions of four CGD patients were analyzed: (1) pus from a gingival abscess present for 48 h (no culture performed), and three samples of chronic inflammatory lesions, which were (2) pus from a subcutaneous abscess of the neck present for 2 weeks (no microorganisms found), (3) tissue sample from a sub-muscular thoracic abscess inflamed for 4 weeks (*Aspergillus fumigatus* cultured), and (4) a lymph node of the neck with inflammation present for 6 weeks (*Burkholderia cepacia* cultured).

**Figure 1:**
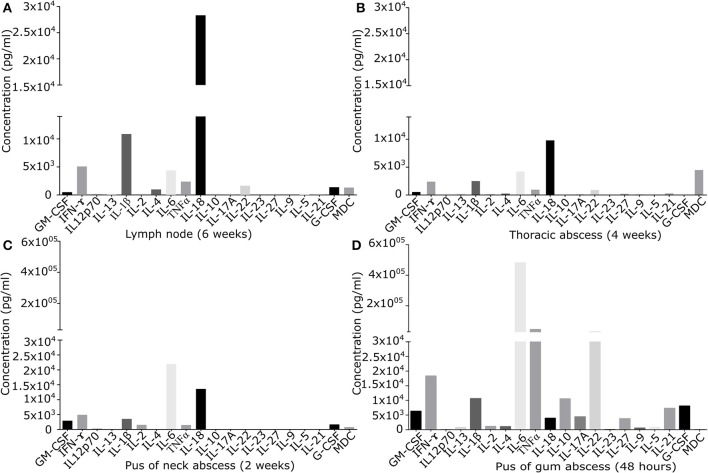
Quantification of signaling molecules in inflamed primary derived human CGD material of **(A)** lymph node of the neck present since 6 weeks, **(B)** sub-muscular thoracic abscess inflamed since 4 weeks, **(C)** pus from subcutaneous abscess of the neck present since 2 weeks, **(D)** puss from gingival abscess present since 48 h. In the supernatant of primary derived smashed tissues signaling molecules were quantified by Multiplex immunoassay. Results are given in pg/ml of local undiluted concentrations. Tissue types and duration of inflammation at the time of the analysis are indicated.

In the extracellular fraction of tissue samples, levels of signaling molecules were quantified (Figure 1) and cellular composition of isolated cell population was analyzed (Table S1).

While granulocytes were the predominant cell type in the acute gingival abscess (87.6% CD15+ cells), in chronically inflamed neck abscess material and thoracic tissue, granulocytes were underrepresented (0.2 and 0.7% CD15+ cells, respectively). CD14+ macrophages were noted in moderate amounts in chronically inflamed thoracic tissue (9.9%), whilst acute gingival abscess and neck abscess contained only 0.4 and 0.7% CD14+ cells, respectively. iNKT cells (CD3, CD56, and vαJ18CD56+) were the predominating cell type (83.9%) in 2 weeks old neck abscess, whereas they accounted for only 3.4% of cells in 4 weeks old thoracic inflammation, and 0.1% in acute gingival abscess. NK cells (CD57+) accounted for only 0.2 and 2.4% in chronically inflamed thoracic and neck samples, and were undetectable in acute gingival abscess. Cytotoxic T cells (CD3/CD8+) were present in thoracic tissue (9.8%) and, in low numbers, in the acute gingival abscess (0.3%), and undetectable in the neck abscess. Helper T cells (CD3/CD4+) were found in low numbers in neck abscess (1.4%), but were absent in thoracic tissue or gingival abscess. Thoracic tissue presented 37.4% CD3(+)CD4(–)CD8(–) double negative T cells (21), which were negative for other analyzed surface markers. B cells were hardly detectable in thoracic tissue (0.1%), and absent in all other tissues.

### Quantification of Released Cytokines/Chemokines

Cytokine/chemokine pattern in acute inflammation (48 h pus) with high amounts of IL-6, TNFα, and IL-22, differed significantly from that found in chronically inflamed tissues. IL-22 and TNFα were detectable only in low quantity in chronically inflamed tissue. IL-6 was detectable in all samples, but in chronically inflamed samples at maximum 10% of the quantity found in pus of acute inflamed tissue containing high percentage of neutrophils. In chronic inflammation in lymph node and thoracic tissue, IL-18 was the predominant cytokine identified. IFN-γ was detectable in all tissues in moderate amount (Figure 1).

Cytokine and chemokine levels detected in inflamed lymph node and in other inflamed tissues were significantly higher than those quantified in a control lymph node (Figure S1). IFN-γ and IL-18 levels in inflamed lymph node tissue exceeded the levels of control tissue by factor 2.6 and 6.3. Of note, local cytokine/chemokine concentrations were not reflected by serum levels: inflamed lymph node tissue expressed several signaling molecules at very low levels (G-CSF, GM-CSF, IL-4, IL-22, MDC), while high levels of IL-18, IL-1β, IFN-γ, IL-6, and TNFα were found (Figure 1A). At the same time, in serum only IFN-γ, TNFα, and IL-18 could be quantified with serum levels ranging between 4.0 and 9.8% of the corresponding local tissue levels. Locally, IL-18 and IFN-γ were present in inflamed lymph node tissue at the levels of 28,328 and 5,081 ng/ml, whilst only low amounts of 227 and 98 ng/ml were detectable in serum.

### Analysis of Chronically Inflamed Lymph Node Tissue

**Figure 2:**
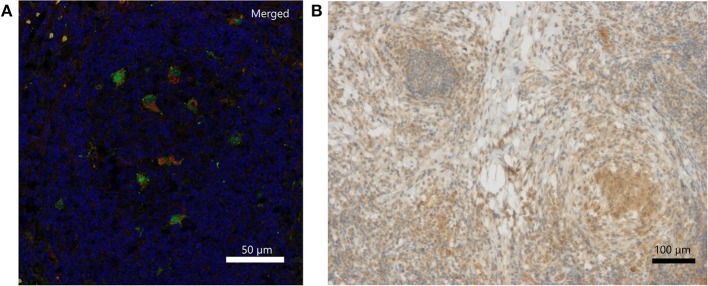
Detection of IL-18 and IFN-γ in inflamed lymph node tissue of a CGD patient at 6 weeks of inflammation. **(A)** Merged DAPI (blue), IL-18 (green)/CD68 (red) double immunofluorescence staining. IL-18 signal (FITC, Alexa488) colocalized with CD68 (Cy3) on tissue macrophages. Single stainings are in Figure S1. **(B)** Immunohistochemical detection of IFN-γ.

Immunohistochemically, IFN-γ was weakly detectable lining up along necrotic tissue and presenting within local clustered structures (Figure 2B). The exact source of IFN-γ is a matter of further investigation. IL-18 staining revealed individual cells, which were clearly positive for IL-18, which is also called IFN-γ-inducing factor. Macrophages were partially positive for IL-18 in the non-necrotic areas and in the germinal cells of the lymph nodes. In contrast, the macrophages forming large granulomas around necrotic tissue were mostly negative for IL-18. Double immunofluorescence staining of inflamed lymph node tissue identified CD68-positive macrophages as one source of IL-18 expression (Figure 2A; Figures S3A–C). Representative pictures of inflamed CGD lymph node tissue upon hematoxylin and eosin (H&E) staining are provided in Figure S2.

### CGD Macrophages Can Acquire M1, M2a, M2b, or M2c Priming Activity

To distinguish between a potential priming defect or bias in CGD macrophages, and a persistent paracrine IL-18/IFN-γ signaling-loop that would keep macrophages in a pro-inflammatory state, the priming capacity of CGD macrophages was analyzed. Blood-derived macrophages of healthy controls and of CGD patients without residual ROS production were either left unstimulated (M0) or were primed to M1, M2a, M2b, or M2c activity. Twenty four hours post priming, obtained macrophage populations were analyzed for surface marker presentation (Table 1; Figure S4) and cytokine release (Table 2; Figure S5).

**Table 1:** Surface marker presentation of CGD macrophages compared to control upon *ex vivo* priming for 24 h.

**Surface marker**	**M0**	**M1**	**M2a**	**M2b**	**M2c**
CD86	CGD 12.3 ± 1.2 CTL 13.0 ± 1.1	CGD 25.9 ± 2.5 CTL 25.9 ± 1.5	CGD 23.1 ± 1.9 CTL 23.6 ± 2.5	CGD 14.2 ± 0.7 CTL 14.0 ± 1.3	CGD 11.8 ± 1.0 CTL 11.6 ± 0.9
CD209	CGD 6.2 ± 1.8 CTL 10.6 ± 0.8	CGD 11.1 ± 3.0 CTL 12.5 ± 2.2	CGD 35.4 ± 5.4 CTL 37.3 ± 7.0	CGD 8.9 ± 1.6 CTL 10.9 ± 2.1	CGD 9.6 ± 2.0 CTL 11.0 ± 1.1
CD206	CGD 34.4 ± 2.9 CTL 29.8 ± 4.9	CGD 32.3 ± 2.7 CTL 27.3 ± 4.8	CGD 63.3 ± 5.0 CTL 62.2 ± 4.9	CGD 19.3 ± 1.2 CTL 18.9 ± 5.0	CGD 38.0 ± 4.1 CTL 29.8 ± 3.9
Mer	CGD 105.9 ± 9.1 CTL 129.9 ± 17.9	CGD 58.7 ± 5.9 CTL 52. 6 ± 12.4	CGD 61.2 ± 4.7 CTL 76.2 ± 11.2	CGD 19.1 ± 0.5 CTL 23.8 ± 4.8	CGD 106.4 ± 16.3^*^ CTL 136.6 ± 10.8^*^
CD163	CGD 12.1 ± 1.7 CTL 15.6 ± 2.0	CGD 7.2 ± 2.4 CTL 8.6 ± 0.6	CGD 9.2 ± 3.6 CTL 10.4 ± 0.3	CGD 3.3 ± 0.6 CTL 3.6 ± 0.5	CGD 26.1 ± 4.9 CTL 28.6 ± 3.8

*Blood monocyte-derived macrophages of CGD patients and healthy controls were primed to macrophage subpopulations for 24 h and analyzed by flow cytometry. Surface marker presentation is expressed as geomean of mean fluorescence intensities of gated positive populations ± SEM. N = 4*,

* *p < 0.05 (bold)*.

**Table 2:** Cytokine/chemokine secretion of CGD macrophages compared to control upon 24 h priming.

**Cytokine**	**M0**	**M1**	**M2a**	**M2b**	**M2c**
IL-18	CGD 710.2 ± 150.1 CTL 646.8 ± 79.4	CGD 4,447.6 ± 241.1 CTL 4,464.2 ± 250.2	CGD 709.2 ± 125.0 CTL 840.6 ± 117.5	CGD 708. 5 ± 62.0 CTL 931.4 ± 145.5	CGD 658.4 ± 101.6 CTL 719.9 ± 97.1
IL-12p70	CGD 72.9 ± 72.9 CTL 0.00 ± 0.0	CGD 747.9 ± 173.6 CTL 978.7 ± 275.3	CGD 6.3 ± 6.3 CTL 90.8 ± 27.3	CGD 11.4 ± 7.9 CTL 2.3 ± 1.4	CGD 0.0 ± 0.0 CTL 0.2 ± 0.2
IL-13	CGD 48.5 ± 33.0 CTL 8.8 ± 8.8	CGD 182.3 ± 24.8 CTL 186.4 ± 29.0	CGD 47.3 ± 31.5 CTL 81.7 ± 30.2	CGD 17.1 ± 17.1 CTL 25.1 ± 25.1	CGD 25.1 ± 25.1 CTL 20.1 ± 20.1
IL-22	CGD 1,688.1 ± 1688.1 CTL 0.0 ± 0.0	CGD 2,995.6 ± 1,002.7 CTL 1,456.8 ± 650. 4	CGD 0.0 ± 0.0 CTL 89.7 ± 89.7	CGD 29.3 ± 29.3 CTL 140.3 ± 140.3	CGD 533.8 ± 234.2 CTL 841.4 ± 408.4
IFN-γ	CGD 295.6 ± 189.3 CTL 126.2 ± 10.3	CGD n.d. CTL n.d.	CGD 250.3 ± 84.3 CTL 250.3 ± 31.8	CGD 196.4 ± 10.4 CTL 206.9 ± 36.0	CGD 130.5 ± 21.7 CTL 155.9 ± 15.6
IL-6	CGD 3,540.2 ± 1942.3 CTL 2,163.3 ± 757.6	CGD 23,594.9 ± 2,157.9^*^ CTL 16,522.7 ± 2,333.0^*^	CGD 2,177.5 ± 1,275.8 CTL 921.3± 402.1	CGD 16,000.2 ± 3,064.7 CTL 18,429.2 ± 2,870.6	CGD 1,189.2 ± 397.8 CTL 995.6 ± 419.0
TNFα	CGD 594.7 ± 272.7 CTL 592.7 ± 207.8	CGD 19,227.8 ± 4,476.1 CTL 27,435.5 ± 9,340.2	CGD 1,052.4 ± 307.5 CTL 1,242.0 ± 136.7	CGD 24,135.0 ± 7,174.5 CTL 36,090.4 ± 12,023.0	CGD 114.2 ± 29.4 CTL 161.0 ± 6.3
IL-5	CGD 168.5 ± 128.4 CTL 592.7 ± 207.8	CGD 19,227.8 ± 4,476.1 CTL 27,435.5 ± 9,340.2	CGD 1,052.4 ± 307.5 CTL 1,242.0 ± 136.7	CGD 24,135.0 ± 7,174.6 CTL 36,090.4 ± 12,023.0	CGD 114.2 ± 29.4 CTL 161.0 ± 6.3
IL-4	CGD 326.2 ± 270.8 CTL 36.7 ± 36.7	CGD 903.1 ±32.6 CTL 900.6 ± 22.6	CGD n.d. CTL n.d.	CGD 565.3 ± 119.3 CTL 759.2 ±127.4	CGD 9.4 ± 9.4 CTL 73.3 ± 42.5
MDC	CGD 3,251.1 ± 900.5 CTL 3,647.2 ± 1,107.1	CGD 6,458.5 ± 935.9^*^ CTL 3,237.7 ± 991.8^*^	CGD 16,608.2 ± 873.5 CTL 13,977.8 ± 1,472.6	CGD 4,286.1 ±681.6 CTL 1,617.5 ± 573.5	CGD 3,545. 2 ± 1,657.2 CTL 1,963.8 ± 675.7
GM-CSF	CGD 1,116.6 ± 830.5 CTL 405.2 ± 211.4	CGD 1,981.0 ± 133.9 CTL 1,981.2 ± 127.9	CGD 7,838.4 ± 351.8 CTL 8,907.1 ± 609.3	CGD 9,012.3 ± 2,624.1 CTL 12,715.1 ± 4,332.3	CGD 188.5 ± 188.5 CTL 423.1 ± 284.7
G-CSF	CGD 16.8 ± 2.6 CTL 10.6 ± 3.0	CGD 37.6 ± 9.0 CTL 32.0 ± 4.8	CGD 13.8 ± 4.3 CTL 11.4 ± 2.6	CGD 887.8 ± 329.5^*^ CTL 571.9 ±103.1^*^	CGD 16.7 ± 7.2 CTL 11.3 ± 2.6
IL-1β	CGD 176.5 ± 68.9 CTL 103.6 ± 28.6	CGD 263.4 ± 22.5 CTL 227.0 ± 25.5	CGD 145.7 ± 22.4 CTL 85.6 ± 26.7	CGD 452.2 ± 178.1 CTL 415.8 ± 105.4	CGD 124.8 ± 16.9 CTL 91.8 ± 27.3

*Blood monocyte-derived macrophages of CGD patients and healthy controls were primed (24 h), and cytokine/chemokine concentrations were quantified in cell culture supernatants. Concentrations are expressed in pg/ml of undiluted tissue as mean ± SEM; N = 4. n.d., not analyzed as it was added to cell culture medium*.

* *p < 0.05 (bold)*.

Unprimed macrophages showed only receptor tyrosine-protein kinase Mer (EC:2.7.10.1) expression and were negative for CD163. M1-primed macrophages showed high expression of CD86 and were CD206-/CD209-. CD206 and CD209 expression is characteristic for M2a, and CD163 expression is characteristic for M2c primed macrophages. None of the analyzed surface markers were presented by *ex vivo* M2b primed macrophages. Statistically significant differences in surface marker presentation of CGD vs. control macrophages were only observed for Mer presentation in M2c macrophages (Table 1).

In M1-primed macrophages, high levels of IL-4, IL-5, IL-6, IL-12p70, IL-13, IL-18, IL-22, and TNFα were detected, of which IL-12p70, IL-13, IL-18, and IL-22 were characteristic for this cell population. M2a-primed macrophages released MDC and GM-CSF in significant quantities, of which MDC was only released by M2a macrophages. M2b macrophages were positive for IL-4, IL-5, IL-6, and TNFα, but in contrast to M1 macrophages which also expressed these cytokines, were negative for IL-12p70, IL-13, IL-18, and IL-22. M2b-primed macrophages released only low amounts of MDC, which was synthesized by M2a-primed macrophages. The release of G-CSF was only observed in M2b macrophages. Of note, none of the cytokine levels derived from M2c-primed macrophages exceeded those of unprimed M0 macrophages. Interestingly, *ex vivo* priming of macrophages was not accompanied by induction of IFN-γ secretion (Table 2; Figure S5). IFN-γ quantification in M1-primed macrophages was not informative, as it was added to cell culture for M1 priming.

Statistically significant differences in the supernatant cytokine and chemokine levels between CGD and control macrophages were not seen for most of the molecules analyzed, except for IL-6 in M1, MDC in M1 and G-CSF in M2b with CGD levels exceeding controls (Table 2). Taken together, we conclude that an obvious inherent priming defect in CGD macrophages was not observed.

### Macrophage Re-priming as Passive Reaction to Their Microenvironment

**Figure 3:**
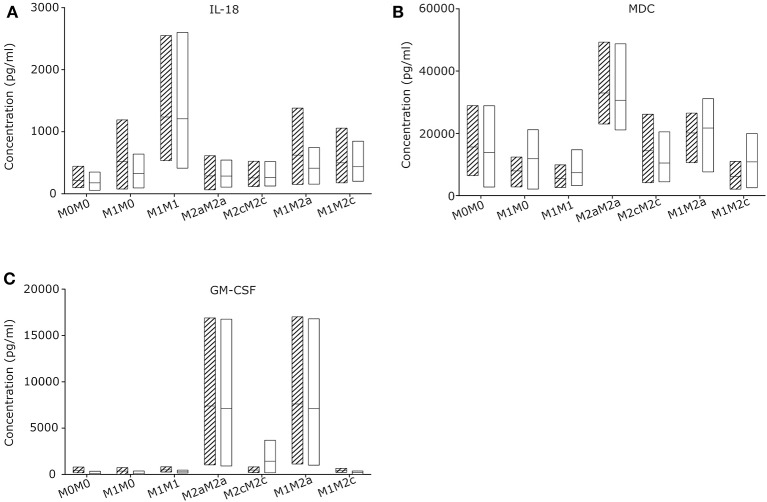
CGD and control macrophage derived cytokines/chemokines upon re-priming (72 h) of M1 primed (24 h) macrophages. Macrophages of CGD patients and of controls were left unstimulated (24 + 72 h) (M0M0), or were primed (24 h), followed by withdrawal of stimulus (M1M0), or by re-priming for 72 h (M1M1; M2aM2a; M2cM2c; M1M2a; M1M2c). Released cytokines/chemokines of **(A)** IL-18, **(B)** MDC and **(C)** GM-CSF were quantified after 24 +72 h. *N* = 3, empty = control, striped = CGD.

To evaluate the macrophage re-priming capacity, CGD and control macrophages were primed first for 24 h to pro-inflammatory M1, followed by 72 h in stimulus-free medium (M1M0) or with medium supplemented with stimuli inducing M2a (M1M2a) or M2c (M1M2c) re-priming followed by quantification of cytokine and chemokine release. Macrophages left unstimulated (M0M0) or exposed to M2a (M2aM2a) or M2c (M2cM2c) stimuli for 24 + 72 h served as controls (Figure 3; Figure S4). Upon 72 h re-priming of *in vitro* generated CGD M1 macrophages, absolute cytokine and chemokine levels of all cytokines declined, while cytokine and chemokine patterns of individual populations remained constant. When 24 + 72 h stimulated with M1-inducing agents, CGD and control M1M1 macrophages secreted high levels of IL-18, IL-12p70, IL-13, IL-6, and low levels of G-CSF, IL-1β, IL-5, and IL-4. When stimulated with M2a-inducing agents, secretion of MDC and GM-CSF remained high at 24 + 72 h, corresponding to the pattern at 24 h. When stimulated with M2c-inducing agents, all cytokines released by macrophages remained on low levels, compared to levels released by other macrophage populations. No statistically significant differences between healthy control and CGD macrophages were observed in this experimental series (Figure S6) and both, healthy control and CGD M1 macrophages could be re-primed to anti-inflammatory M2a phenotype by a shift to M2a stimulus for 72 h (M1M2a), judged by GM-CSF level (Figure 3C).

Interestingly, all cytokine levels which were secreted by macrophages upon 24 h of M1 priming, including IL-18, dropped after 72 h of incubation in the absence of stimulation (Figure S4), suggesting that the absence of continuous stimulation leads to conversion into a silent M0 macrophage subpopulation. Our results suggest that the presence of IL-18 releasing macrophages in chronically inflamed CGD lymph node tissue is most likely caused by a local pro-inflammatory microenvironment.

## Discussion

Chronic granulomatous disease (CGD) is a rare immunodeficiency affecting about 1 in 200,000 to 1 in 250,000 life births in Europe per year (4). Statistically, every CGD patient though under prophylactic treatment experiences 0.71 episodes of infection per year including surgical removal of inflamed tissue once in 5 years per patient (37). Therefore, primary-derived patient material is extremely rare. Furthermore, inflammation in CGD can be caused by various pathogens including *Aspergillus, Burkholderia, Nocardia, Serratia*, and *Staphylococcus* (38). These infections manifest most frequently in lung, but can also affect liver, lymph nodes, bone, skin, and soft tissues (38). Furthermore, hyperinfammation in CGD is also reported without detectable pathogen (20). The heterogenity of the causes and the tissue localization is amplified by the kinetics of inflammation. In addition, taking the rarity of the disease and of infection episodes into account, it is almost impossible to obtain homogeneous sample collections of inflamed tissue samples from CGD patients. Accordingly, the samples characterized here represent the general heterogeneity of inflammatory manifestations.

Besides susceptibility to bacterial and fungal infection, chronic hyper-inflammation leading to granuloma formation in internal organs is a characteristic of CGD (39). Both processes are likely linked through impaired ROS formation, concomitant impaired neutrophil extracellular trap (NET) formation and impaired pathogen killing, as well as the impaired NADPH oxidase-dependent oxidation of PS in granulocytic membranes (30, 31, 40, 41).

On the one hand, in neutrophils of healthy individuals NADPH oxidase-derived ROS was reported to be essential for PS oxidation and exposure during phorbol 12-myristate 13-acetate (PMA)-induced cell death (42). Fernandez-Boyanapalli et al., hypothesized that in CGD impaired PS exposure on dying cells results in defective macrophage programming to efferocytosing alternatively-activated macrophages, which in consequence could lead to inefficient clearance of the infection site from dying neutrophils and to enhanced inflammation (32). The inefficient efferocytotic murine macrophage activity can be reversed by IL-4 (32). On the other hand, in the zymosan-induced peritonitis model in CGD mice, the percent of neutrophils exposing PS was only slightly diminished compared to the percentage in WT mice (43). *Ex vivo*, PS exposure by human CGD granulocytes was reported upon spontaneous apoptosis after 24 h of culture (31). In a review, a potential involvement of cytosolic cytochrome C in lipid oxidation in apoptotic cells was discussed (44). Such a mechanism potentially can explain the observed PS oxidation observed *in vivo* in CGD.

Therefore, we propose that in CGD the crucial positive-feedback loop consisting of the secretion of anti-inflammatory IL-4 by macrophages does not take place, and pro-inflammatory M1 macrophages cannot develop into anti-inflammatory M2a macrophages, as this step requires exposure to anti-inflammatory cytokines such as IL-4 and IL-13 (45, 46). To our knowledge, no data regarding the cytokine expression in human primary-derived inflamed CGD patient tissues have been reported so far.

Our results support this hypothesis by showing high levels of IL-18 in chronically inflamed CGD tissue, while IL-6 was dominating in acute phase of inflammation in CGD tissue, most likely synthesized by granulocytes as most frequent cell population in this tissue type (Figures 1A,D). In our hands, IL-18 was released in high quantity by *ex vivo* primed pro-inflammatory M1 macrophages, with CGD macrophages differing from healthy controls only in absolute values of three cytokines (Table 2) and in one surface marker (Table 1). This is in contrast to the report on human CGD macrophages that were described to show impaired production of IL-6, IFN-γ, IL-12p70, IL-13, MCP-1, and MIP-1β, and increased secretion of IL-10 (47). Likely, these differences can be attributed to the fact that these latter data were obtained from monocyte-derived macrophages from CGD patients receiving anti-inflammatory and immunosuppressive treatment, e.g. corticosteroids. In our analysis however, treatment with corticosteroids was an exclusion criterion, as we wanted to exclude potential bias caused by this treatment.

In three CGD tissue samples with inflammation persisting for 2, 4, and for 6 weeks, we could detect IL-18, indicating the persistence of IL-18 releasing cells. In inflamed CGD lymph node tissue, macrophages were one source of IL-18 secretion. The presence of macrophages in our CGD chronically inflamed tissue is in line with the histological description of granulomas in CGD colitis (48). In murine X-CGD, the presence of pro-inflammatory M1 macrophages has been reported, whilst WT mice showed the presence of anti-inflammatory M2 macrophages (32, 33). Our *ex vivo* experiments however clearly showed that CGD macrophages have the capacity to acquire all priming activities, provided they are exposed to the appropriate stimuli, and that withdrawal of M1 priming stimulus weakens their pro-inflammatory activity. Hence, in inflamed CGD lymph node tissue, the persistence of IL-18 secreting macrophages might be explained by a persisting local microenvironment that keeps macrophages in M1 state and leads to a persistent pro-inflammatory milieu.

IL-18, originally called *IFN-*γ *inducing factor*, is known to enhance the secretion of IFN-γ from NK cells and CD4+ T helper 1 lymphocytes (49). It has been shown that in combination with IL-15, IL-12, or with allergens IL-18 enhances, or synergistically induces the production of IFN-γ in cord blood mononuclear cells (50) or in NK cells (51). IFN-γ in turn may prime macrophages to pro-inflammatory M1 phenotype when combined with bacterial LPS (28). Indeed, we found increased levels of IFN-γ in chronically inflamed lymph node tissue by immunohistochemistry and cytokine quantification. Further investigation is needed to prove that signaling emerging from IL-18 and IFN-γ secreting cells build a paracrine signaling loop *in vivo*. We were however unable to formally prove the presence of IFN-γ releasing NK and/or T cells in the limited amount of samples analyzed. The initial trigger for inflammation would be an infectious organism or an infammasome stimulus, leading to neutrophil recruitment and persistent inflammatory cytokine secretion by the latter in the absence of NET formation. Tissue macrophages would thus be primed into M1 pro-inflammatory state without the possibility to return into neutral M0, or anti-inflammatory M2a state, leading to increased IL-18 secretion. On the one hand, a hypothetical IL-18 and IFN-γ signaling loop could contribute to attraction of NK and/or T-cells and to an increased IFN-γ production. This scenario is supported by the fact, that clinically CGD patients might develop macrophage activation syndrome leading to hemophagocytosis, similar to hereditary hemophagocytic lymphohistiocytosis (52, 53). On the other hand, IFN-γ has been used for years in many patients to reduce infectious triggers for inflammation, with no evidence of increased hyper-inflammatory complications. Therefore, it is likely that IL-18 and IFN-γ axis is one out of several factors potentially contributing to hyperinflammation in CGD.

## Data Availability Statement

All datasets generated for this study are included in the manuscript/Supplementary Files.

## Author Contributions

Under the supervision of US and JR, VM designed the experiments, analyzed the results, and wrote the manuscript. The human tissue samples presented in Figure 2 were processed and stained by MD and Sophistolab (Muttenz, Switzerland). RV and OP took the pictures used for Figure 2. VM performed all the other experiments and prepared the figures. AD, JE, UCS, SH, CS, SE and JR provided clinical data and patient material. MD performed histological analyses and revised the manuscript.

### Conflict of Interest

The authors declare that the research was conducted in the absence of any commercial or financial relationships that could be construed as a potential conflict of interest.

## Supplementary Material

Figure S1 Cytokine and chemokine levels quantified in inflamed lymph node vs. in control lymph node tissue. In primary derived control lymph node tissue **(B)** and in a chronically inflamed lymph node tissue of a CGD patient **(A)**, signaling molecules were quantified by Multiplex immunoassay. Results are given in pg/ml of local undiluted concentrations. Tissue types and duration of inflammation at the time of the analysis are indicated.

Figure S2 Hematoxylin and eosin (H&E) staining. Inflamed lymph node tissue of a CGD patient with inflammation persisting for 6 weeks was paraffin-embedded. Sections of embedded lymph node tissue were stained with H&E according to standard protocol as described recently (54). Images were then analyzed with a bright field microscope (Axiovert S100TV, Carl Zeiss Vision Swiss AG, Feldbach, Switzerland). Magnification is indicated.

Figure S3 Immunofluorescence microscopy analysis of lymph node tissue of a CGD patient with inflammation persisting for 6 weeks. Paraffin embedded lymph node tissue section was dewaxed and double stained for IL-18 and CD68 and DAPI. Immunofluorescence signal of IL-18 (FITC) was amplified by tertiary staining with an anti-FITC Alexa488 antibody. CD68 is visualized by Cy3. Individual pictures detecting DAPI **(C)**, IL-18 **(B)** and CD68 (Cy3) **(A)** are shown. Merged picture is shown in Figure 2A Scale bar: 50 μm.

Figure S4 Surface marker presentation **(A)** CD86, **(B)** CD209, **(C)** CD206, **(D)** Mer and **(E)** CD163, on healthy control and CGD macrophages upon *ex vivo* priming for 24 h. Blood monocyte-derived macrophages of CGD patients and of healthy controls were primed to macrophage subpopulations for 24 h and analyzed by flow cytometry analysis. Surface marker presentation is expressed as mean fluorescence intensities of gated positive populations ± standard error of the mean. *N* = 4, ^*^*p* < 0.05, striped box-plots, CGD; empty box-plots, control. Values are given in Table 1.

Figure S5 Cytokine/chemokine secretion of CGD blood monocyte derived macrophages compared to control upon 24 h priming. Blood monocyte derived macrophages of CGD patients and of healthy controls were primed for 24 h and cytokine/chemokine concentrations in cell culture supernatants were quantified by immunoassay. **(A)** IL-18, **(B)** IL-12p70, **(C)** IL-13, **(D)** IL-22, **(E)** IFN-γ, **(F)** IL-6, **(G)** TNFα, **(H)** IL-5, **(I)** IL-4, **(J)** MDC, **(K)** GM-CSF, **(L)** G-CSF, **(M)** IL-1β. Concentrations are expressed in Box-plot diagrams representing maximum values, means, minimum values in pg/ml and statistical analyses of CGD vs. controls. Striped box-plots, CGD; empty box-plots, control. *N* = 4, ^*^*p* < 0.05, n.d., not done as cytokines were utilized for cell stimulation. ^*^*p* < 0.05. Values are given in Table 2.

Figure S6 Similar cytokine secretion of 24 h primed CGD macrophage subpopulations followed by additional 72 h priming compared to healthy control macrophages. Monocyte-derived macrophages of CGD patients and healthy controls remained unstimulated (M0) or were primed to M1, M2a, or M2c followed by additional 72 h either unstimulated (M0) or primed to M1, M2a, or M2c as described. Cytokines released into the supernatants were quantified by immunoassay. IL-18, MDC and GM-CSF are shown in Figure 3. **(A)** IL-12p70, **(B)** IL-13, **(C)** IL-22, **(D)** IFN-γ, **(E)** IL-6, **(F)** TNFα, **(G)** IL-5, **(H)** IL-4, **(I)** G-CSF, **(J)** IL-1β, **(K)** IL-23, **(L)** IL-9. Box-plot diagrams representing maximum values, means and minimum values of CGD vs. controls are shown. striped box-plots, CGD; empty box-plots, control. *N* = 3, n.d., not done as cytokines were utilized for cell stimulation.

Table S1 Cellular composition of analyzed primary derived CGD patient tissue samples.

## Abbreviations

CGD: chronic granulomatous disease
DAPI, 4′: 6-Diamidin-2-phenylindol
DNA: deoxyribonucleic acid
EDTA: ethylenediaminetetraacetic acid
GI: gastrointestinal
HSCT: hematopoietic stem cell transplantation
LPS: lipopolysaccharide
MNCs: mononuclear cells
NADPH: nicotinamide adenine dinucleotide phosphate
NET: neutrophil extracellular trap
NLRP3: Nod-Like Receptor Pyrin 3
PBS: phosphate buffered saline
PFA: paraformaldehyde
PMA: phorbol 12-myristate 13-acetate
PMNs: polymorphonuclear leukocytes
PPAR-γ: proliferator-activated receptor γ
PS: phosphatidylserine
ROS: reactive oxygen species
SEM: standard error of the mean
WT: wild type.
