# Supplementary figures and images for: High Levels of IL-18 and IFN-γ in Chronically Inflamed Tissue in Chronic Granulomatous Disease
Source: Front Immunol. 2019 Oct 18;10:2236. doi: 10.3389/fimmu.2019.02236 (PMC6813411; doi:10.3389/fimmu.2019.02236)

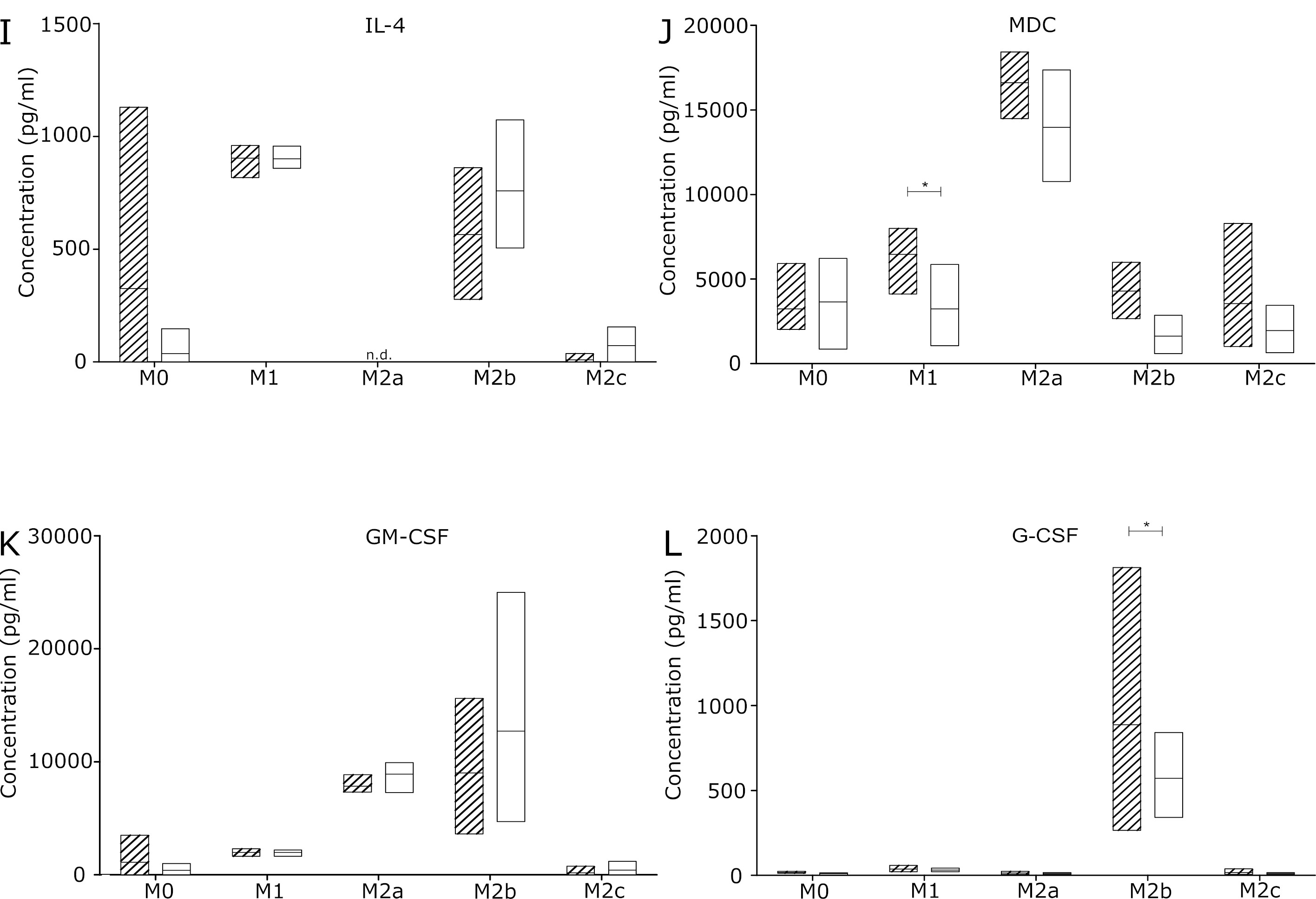

Supplement: Supplementary file 1 [file Image_7.JPEG]

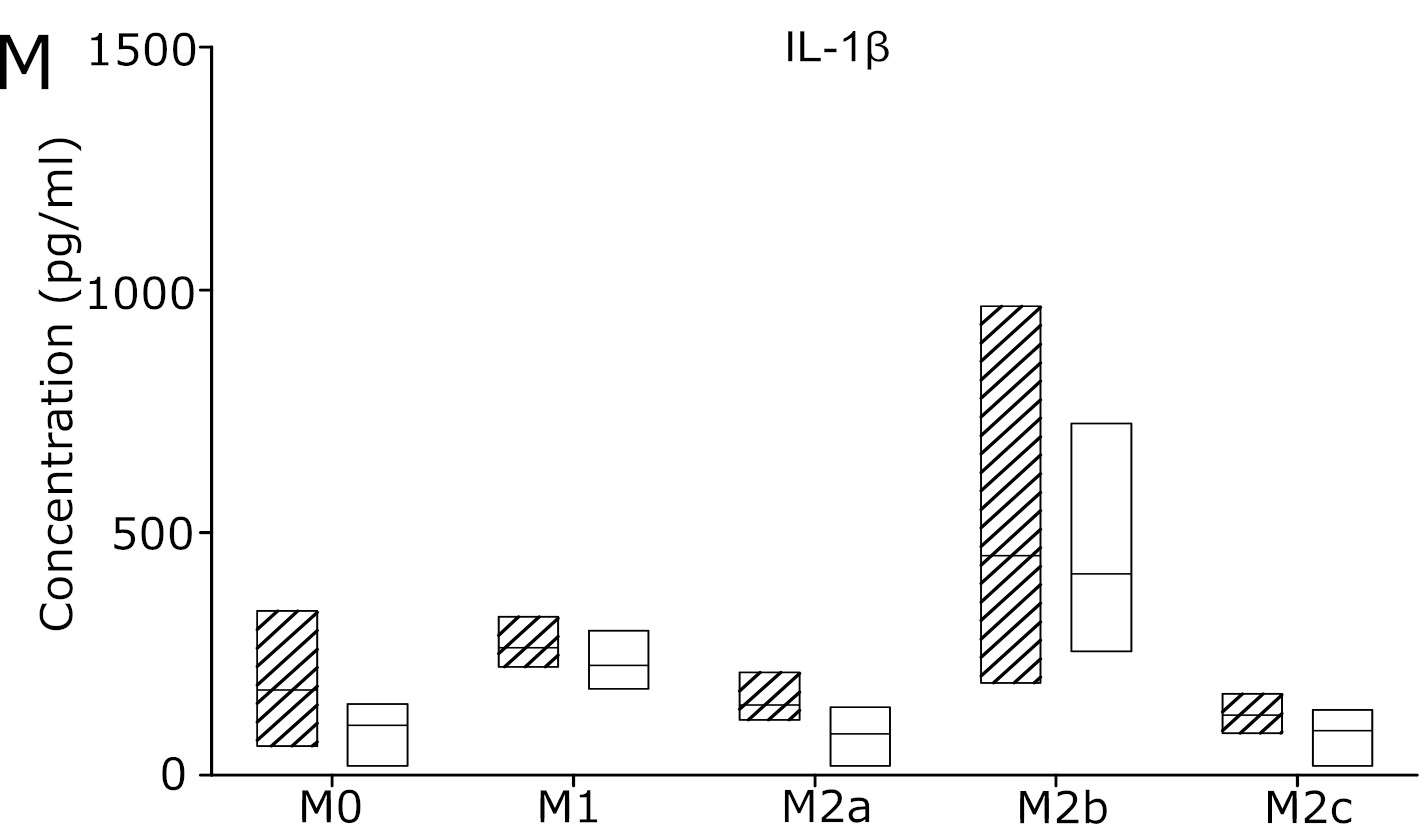

Supplement: Supplementary file 2 [file Image_8.JPEG]

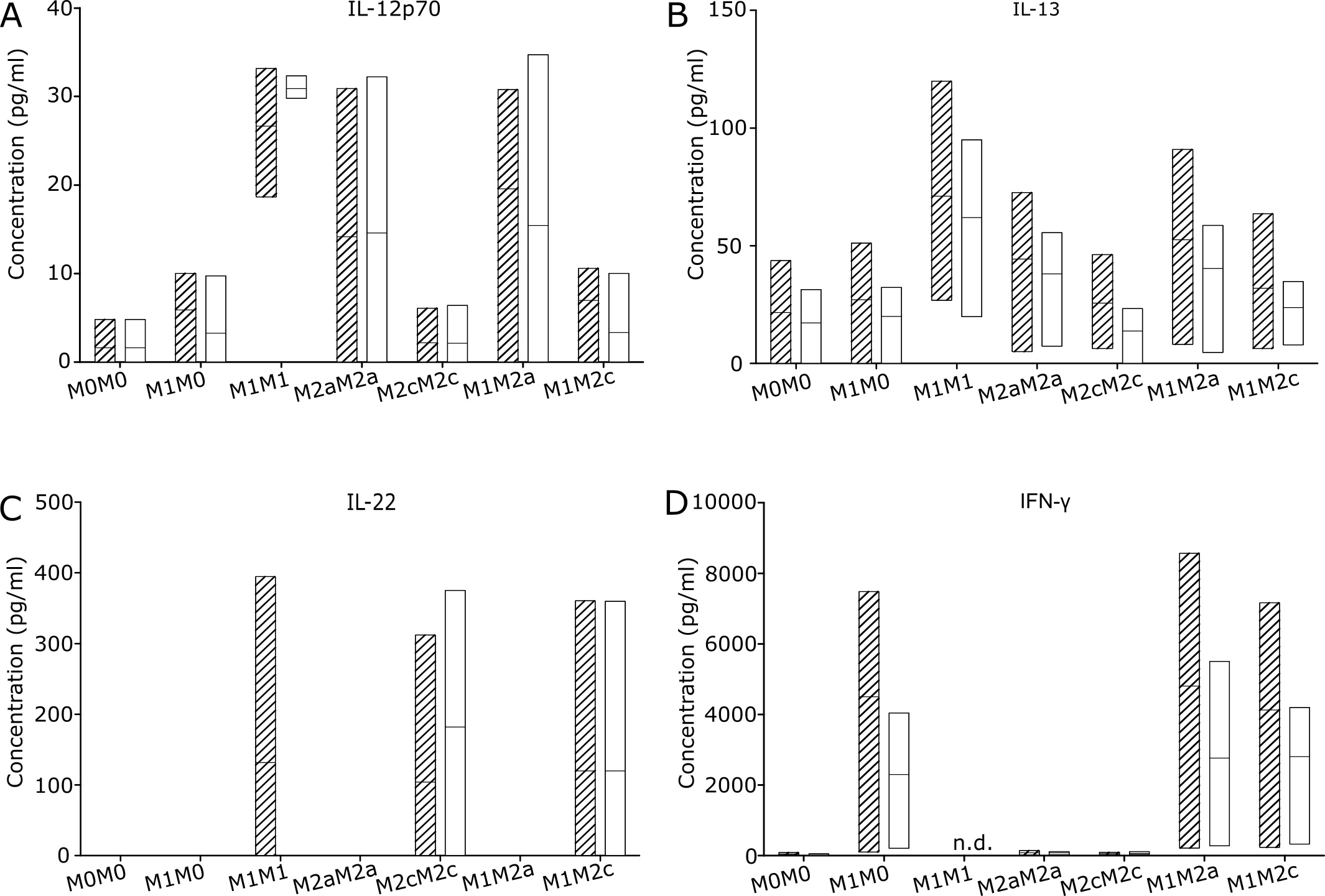

Supplement: Supplementary file 3 [file Image_9.JPEG]

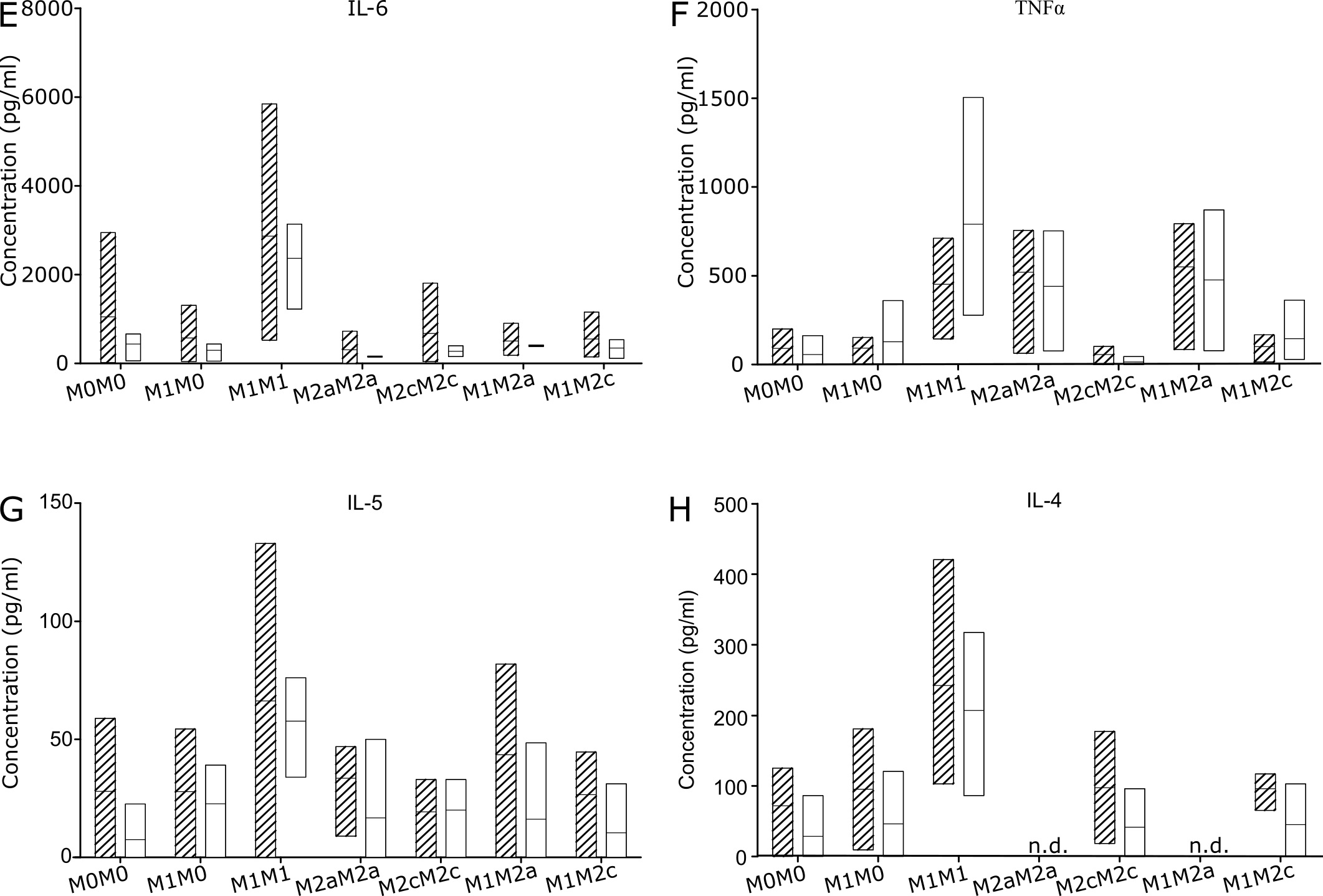

Supplement: Supplementary file 4 [file Image_10.JPEG]

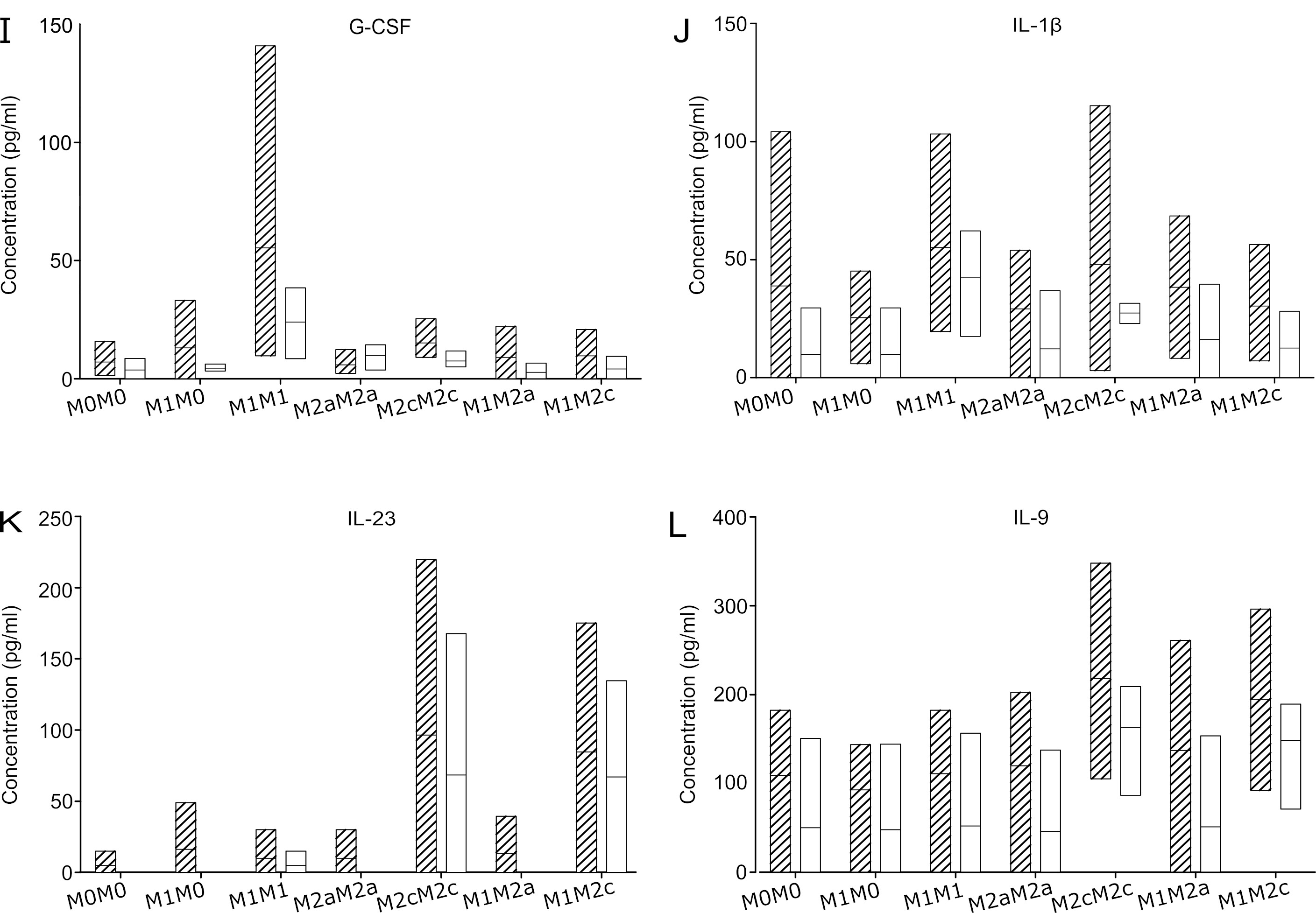

Supplement: Supplementary file 5 [file Image_11.JPEG]

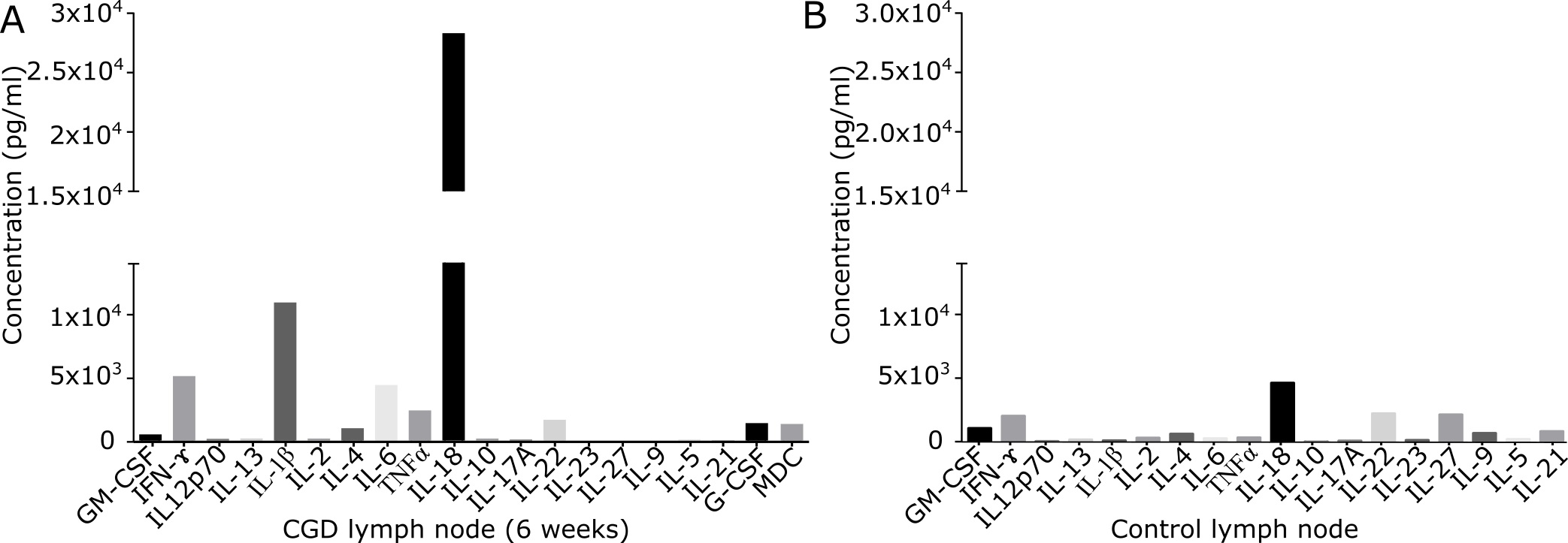

Supplement: Figure S1 — Cytokine and chemokine levels quantified in inflamed lymph node vs. in control lymph node tissue. In primary derived control lymph node tissue (B) and in a chronically inflamed lymph node tissue of a CGD patient (A), signaling molecules were quantified by Multiplex immunoassay. Results are given in pg/ml of local undiluted concentrations. Tissue types and duration of inflammation at the time of the analysis are indicated. [file Image_1.JPEG]

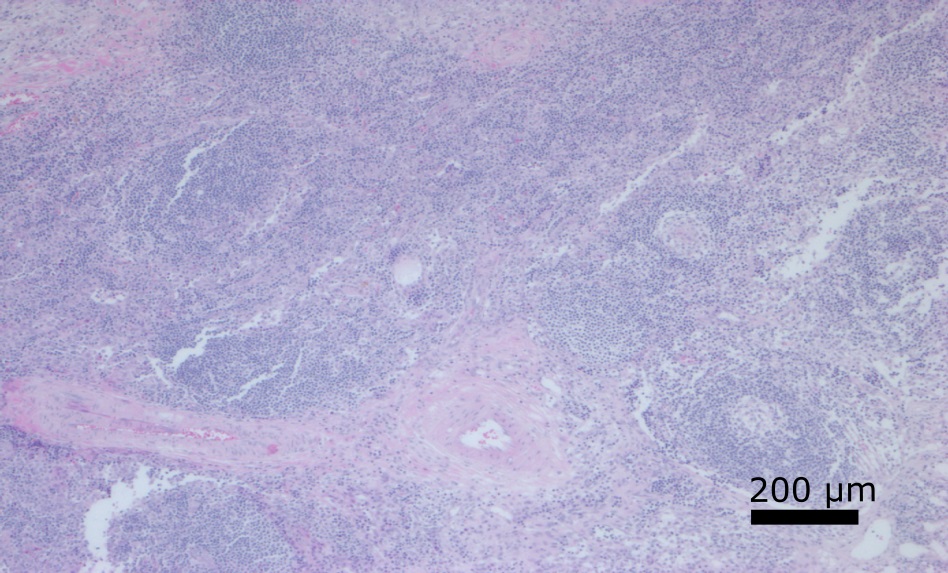

Supplement: Figure S2 — Hematoxylin and eosin (H&E) staining. Inflamed lymph node tissue of a CGD patient with inflammation persisting for 6 weeks was paraffin-embedded. Sections of embedded lymph node tissue were stained with H&E according to standard protocol as described recently (54). Images were then analyzed with a bright field microscope (Axiovert S100TV, Carl Zeiss Vision Swiss AG, Feldbach, Switzerland). Magnification is indicated. [file Image_2.JPEG]

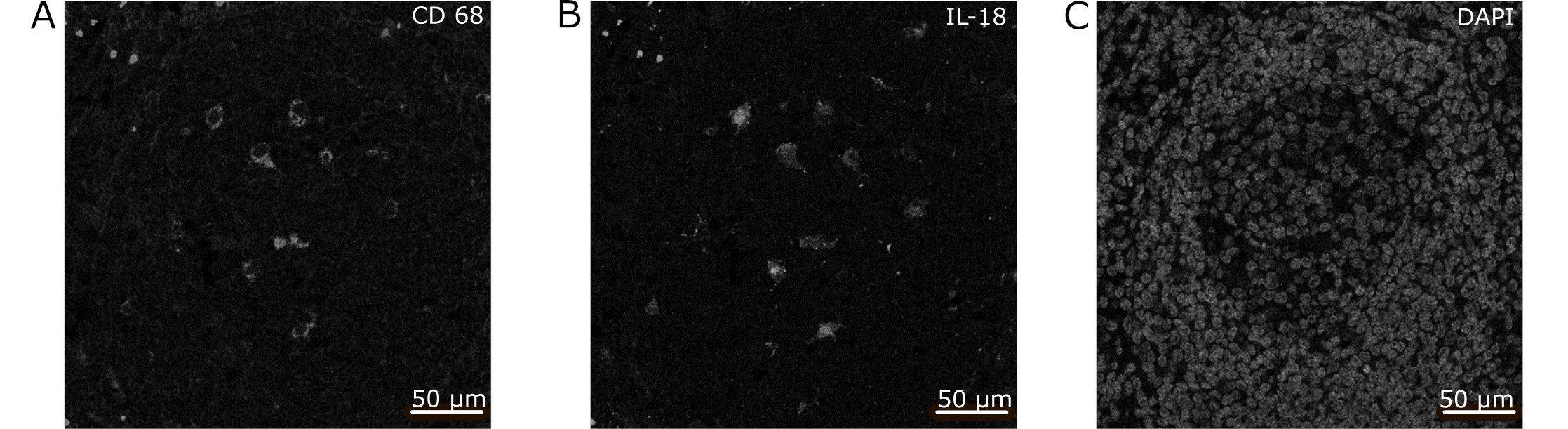

Supplement: Figure S3 — Immunofluorescence microscopy analysis of lymph node tissue of a CGD patient with inflammation persisting for 6 weeks. Paraffin embedded lymph node tissue section was dewaxed and double stained for IL-18 and CD68 and DAPI. Immunofluorescence signal of IL-18 (FITC) was amplified by tertiary staining with an anti-FITC Alexa488 antibody. CD68 is visualized by Cy3. Individual pictures detecting DAPI (C), IL-18 (B) and CD68 (Cy3) (A) are shown. Merged picture is shown in Figure 2A Scale bar: 50 μm. [file Image_3.JPEG]

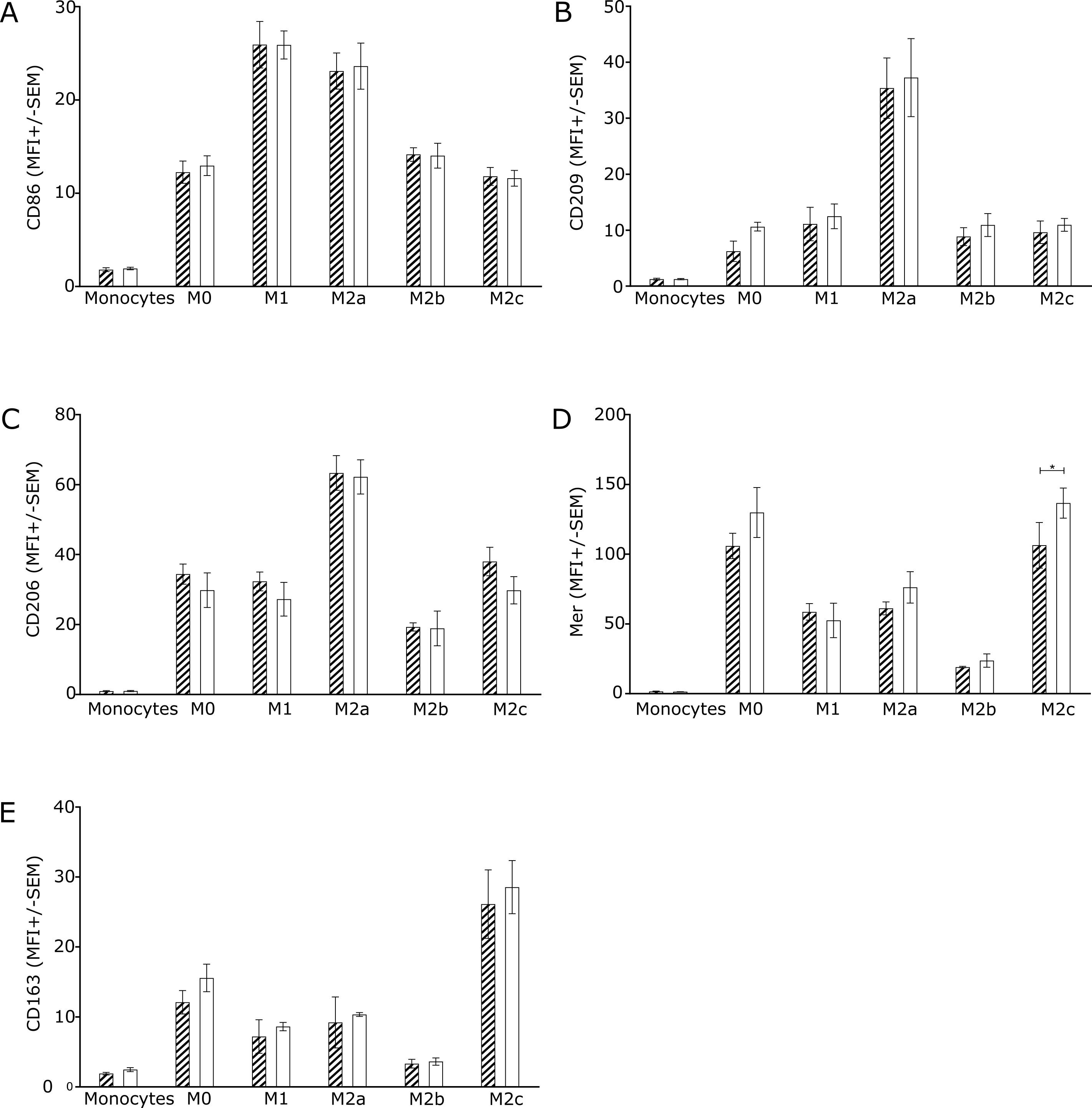

Supplement: Figure S4 — Surface marker presentation (A) CD86, (B) CD209, (C) CD206, (D) Mer and (E) CD163, on healthy control and CGD macrophages upon ex vivo priming for 24 h. Blood monocyte-derived macrophages of CGD patients and of healthy controls were primed to macrophage subpopulations for 24 h and analyzed by flow cytometry analysis. Surface marker presentation is expressed as mean fluorescence intensities of gated positive populations ± standard error of the mean. N = 4, *p < 0.05, striped box-plots, CGD; empty box-plots, control. Values are given in Table 1. [file Image_4.JPEG]

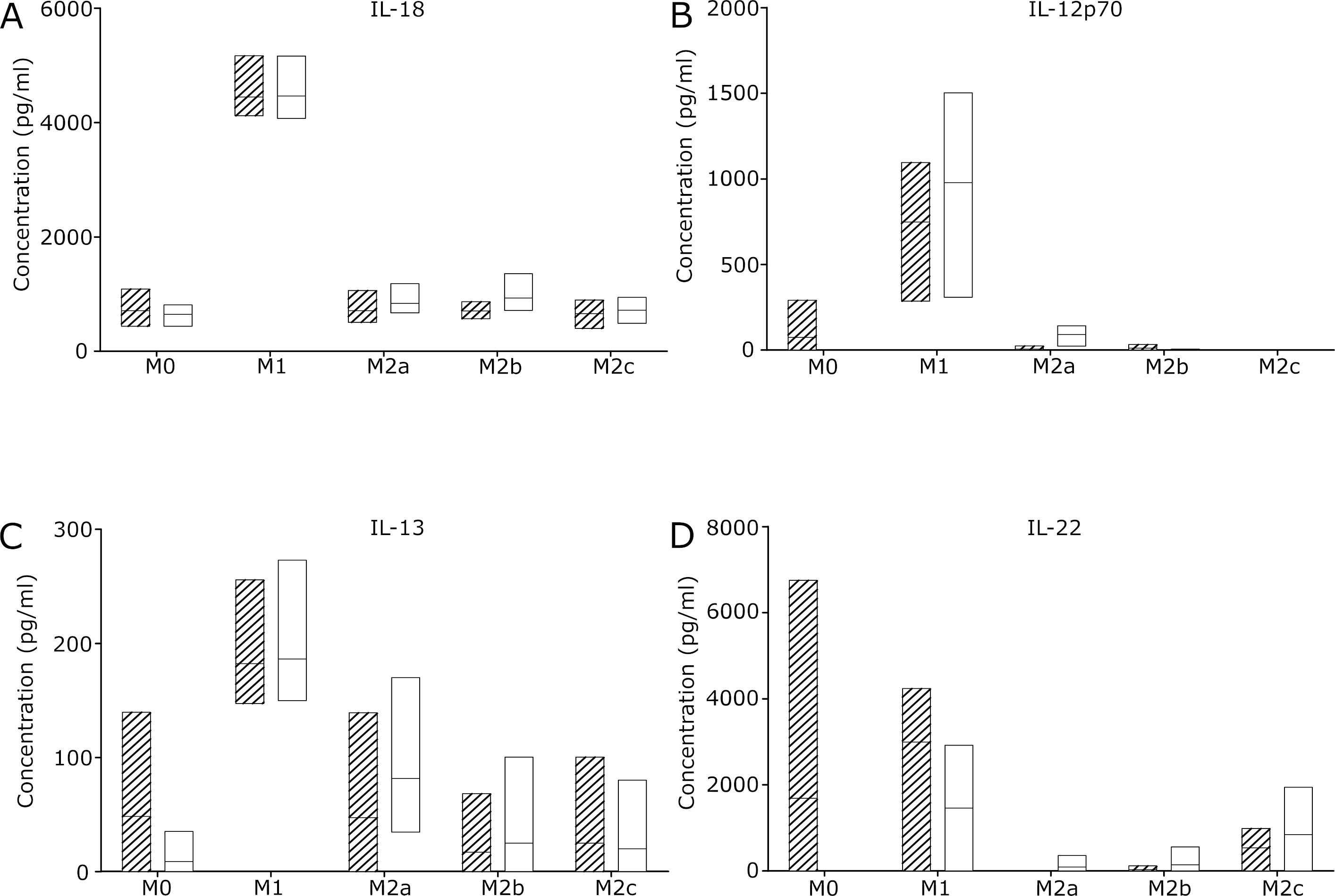

Supplement: Figure S5 — Cytokine/chemokine secretion of CGD blood monocyte derived macrophages compared to control upon 24 h priming. Blood monocyte derived macrophages of CGD patients and of healthy controls were primed for 24 h and cytokine/chemokine concentrations in cell culture supernatants were quantified by immunoassay. (A) IL-18, (B) IL-12p70, (C) IL-13, (D) IL-22, (E) IFN-γ, (F) IL-6, (G) TNFα, (H) IL-5, (I) IL-4, (J) MDC, (K) GM-CSF, (L) G-CSF, (M) IL-1β. Concentrations are expressed in Box-plot diagrams representing maximum values, means, minimum values in pg/ml and statistical analyses of CGD vs. controls. Striped box-plots, CGD; empty box-plots, control. N = 4, *p < 0.05, n.d., not done as cytokines were utilized for cell stimulation. *p < 0.05. Values are given in Table 2. [file Image_5.JPEG]

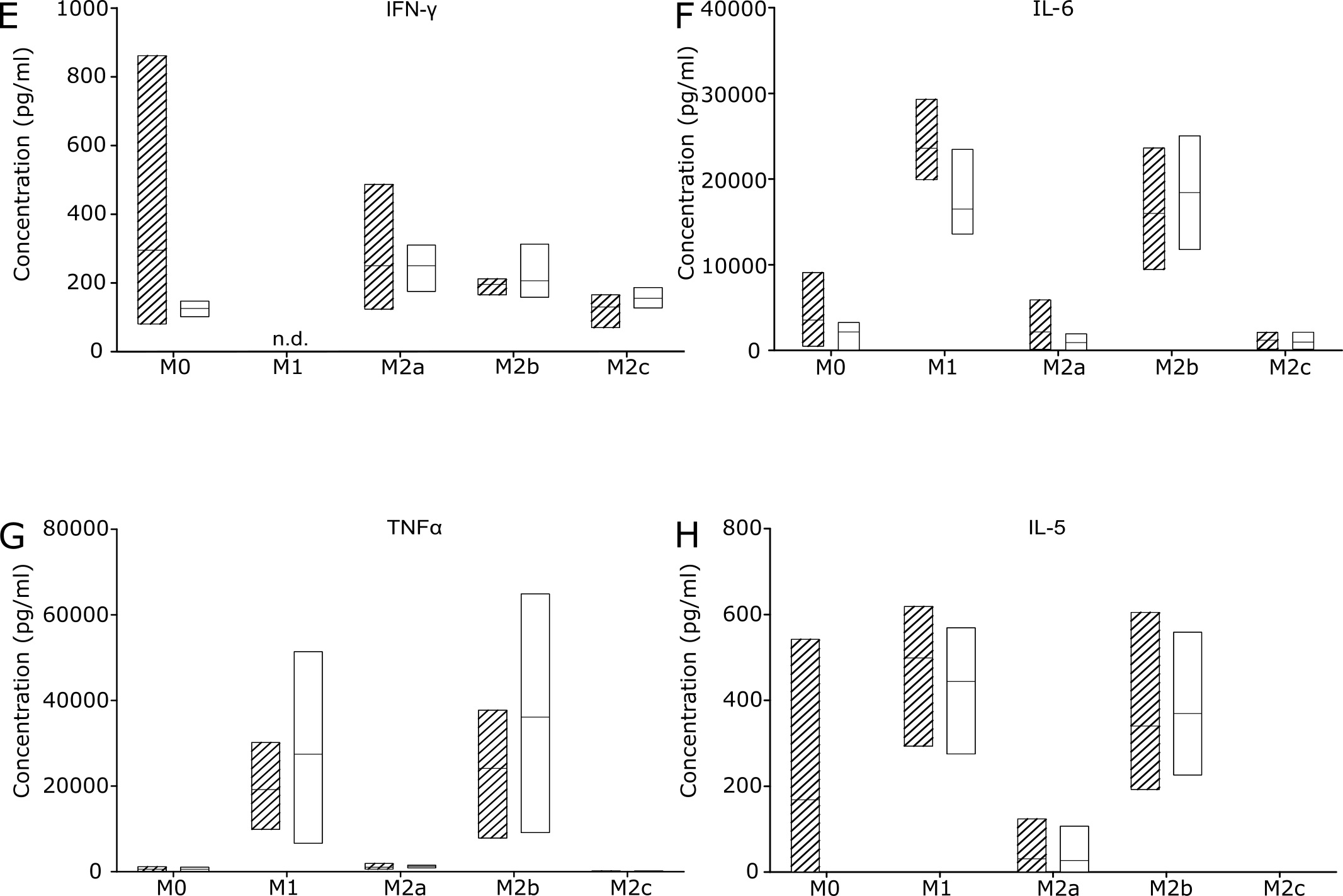

Supplement: Figure S6 — Similar cytokine secretion of 24 h primed CGD macrophage subpopulations followed by additional 72 h priming compared to healthy control macrophages. Monocyte-derived macrophages of CGD patients and healthy controls remained unstimulated (M0) or were primed to M1, M2a, or M2c followed by additional 72 h either unstimulated (M0) or primed to M1, M2a, or M2c as described. Cytokines released into the supernatants were quantified by immunoassay. IL-18, MDC and GM-CSF are shown in Figure 3. (A) IL-12p70, (B) IL-13, (C) IL-22, (D) IFN-γ, (E) IL-6, (F) TNFα, (G) IL-5, (H) IL-4, (I) G-CSF, (J) IL-1β, (K) IL-23, (L) IL-9. Box-plot diagrams representing maximum values, means and minimum values of CGD vs. controls are shown. striped box-plots, CGD; empty box-plots, control. N = 3, n.d., not done as cytokines were utilized for cell stimulation. [file Image_6.JPEG]
