# Supplementary material for: High Levels of IL-18 and IFN-γ in Chronically Inflamed Tissue in Chronic Granulomatous Disease
Source: Front Immunol. 2019 Oct 18;10:2236. doi: 10.3389/fimmu.2019.02236 (PMC6813411; doi:10.3389/fimmu.2019.02236)
Supplement: Table S1 — Cellular composition of analyzed primary derived CGD patient tissue samples. [file Table_1.docx]

**Supplementary Table 1**

Cellular composition of analyzed primary derived CGD patient tissue

| Percent positive cells | | acute gingival abscess | from a sub-cutaneous abscess of the neck | sub-muscular thoracic abscess |
| --- | --- | --- | --- | --- |
|  | Duration of inflammation | 48h | 2 weeks | 4 weeks |
| Surface Marker | Cell |  |  |  |
| CD14 | Monocytes/Macrophages | 0.7 | 0.4 | 9.9 |
| CD15 | Granulocytes | 87.6 | 0.2 | 0.7 |
| CD57 | NK cells | 0 | 2.4 | 0.2 |
| CD56 | NK cells | 0.2 | 83.9 | 2.9 |
| CD3  CD3+CD8+  CD3+CD4+  CD3+vαJα18+CD56+ | T cells  T cells  iNKT cells | 0.4  0.3  0  0.1 | 85.3  0  1.4  83.9 | 50.6  9.8  0  3.4 |
| CD4 | T cells | 0 | 1.4 | 0.3 |
| CD19 | B cells | 0 | 0 | 0.1 |
